# Supplementary material for: Far and Near Contrast Sensitivity and Quality of Vision with Six Presbyopia Correcting Intraocular Lenses
Source: J Clin Med. 2022 Jul 17;11(14):4150. doi: 10.3390/jcm11144150 (PMC9316344; doi:10.3390/jcm11144150)

Supplementary File S1. Quality of vision questionnaire

(*This questionnaire, as displayed here, has been translated from Spanish to English for the purposes of this publication*)

1. Please describe how often do you need to use spectacles to perform far vision tasks

Never	Sometimes	Always

2. Please describe how often do you need to use spectacles to perform intermediate vision tasks (about 60-70 cm)

Never	Sometimes	Always

3. Please describe how often do you need to use spectacles to perform near vision tasks (about 30-50 cm)

Never	Sometimes	Always

4. Please describe the intensity and discomfort of the halos you experience at night (please refer to the image to understand what halos are; 1 is low and 3 is high intensity and or discomfort)

None	1	2	3

5. Please describe the intensity and discomfort of the glare you experience at night (please refer to the image to understand what glare is; 1 is low and 3 is high intensity and or discomfort)

None	1	2	3

Halos

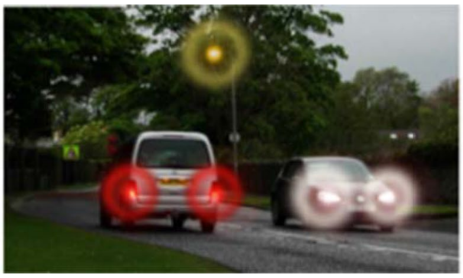

Glare

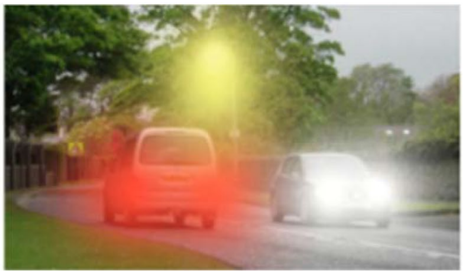

Supplement: Supplementary file 1 [file jcm-11-04150-s001.zip › jcm-1794577-supplementary.pdf]
